# The complete chloroplast genome sequence of *Asarum sieboldii* Miq. (Aristolochiaceae), a medicinal plant in Korea

**DOI:** 10.1080/23802359.2018.1424577

**Published:** 2018-01-12

**Authors:** Chae Eun Lim, Sang-Choon Lee, Soonku So, Su-Min Han, Ji-Eun Choi, Byoung-Yoon Lee

**Affiliations:** a National Institute of Biological Resources, Incheon, Korea;; b Departement of Phylogenomics, Phyzen Genomic Institute, Seongnam, Korea;; c National Park Research Institute, Gangwon-do, Korea

**Keywords:** *Asarum sieboldii*, medicinal plant, chloroplast genome, Aristolochiaceae

## Abstract

*Asarum sieboldii* is a medicinal plant belonging to the Aristolochiaceae family. In this study, complete chloroplast genome sequence of *A. sieboldii* was characterized through *de novo* assembly with next generation sequencing data. The chloroplast genome is 193,356 bp long and has the stereotypical tripartite organization consisting of large single copy region and a pair of inverted repeats. The genome contains 78 protein-coding genes, 30 rRNA genes, and 4 tRNA genes. Phylogenetic analysis revealed that *A. sieboldii* has close relationship with *Piper coenoclatum* (Piperaceae, Piperales).

The genus *Asarum* L. (Aristolochiaceae), which consists of approximately 100 species, is usually distributed in temperate regions of Northern Hemisphere, with the center of diversity in Eastern Asia (Oh 2007). Since ancient times, the dried root of *Asarum* species is a widely used drug in traditional medical practices in China, Korea, and Japan (Yamaki et al. 1996). In particular, *A. sieboldii* called “Seshin” in Korean, are used as remedies for aphthous stomatitis, toothache, gingivitis in Korea and China (Zhou 1993). Although DNA barcode regions (ITS, *rbc*L, and *mat*K) of Korean *Asarum* species have been sequenced and analyzed for species identification and discrimination in genus *Asarum* (National Institute of Biological Resources (NIBR) 2012), the criteria for delimiting the species remain still unclear.

In this study, we determined the chloroplast genome of *A*. *sieboldii* to contribute to the classification and development of DNA markers for authentication of *Asarum* species. The specimen was collected from Mt Deogyu, South Korea (35°51′46.5″ N, 127°44′40.2″ E) and deposited at NIBR herbarium (KB) with the accession number NIBR-VP0000640510. Genome was sequenced using the Illumina MiSeq platform (Illumina Inc., San Diego, CA, USA) and high-quality paired-end reads of ca. 1.7 Gb were used to assemble chloroplast genome (GenBank Accession no. MG551543), as described previously (Kim et al. 2015).

The chloroplast genome was 193,356 bp in length with 36.2% overall GC content. Except the chloroplast genome of *Pelargonium* ×* hortorum* (Chumley et al. 2006), the chloroplast of *A*. *sieboldii* is the largest terrestrial plant chloroplast genome reported to date. In addition, the genome of *A. sieboldii* has the stereotypical chloroplast tripartite organization featuring two copies (IRa and IRb) of a 48,401bp IR separating an LSC region of 96,554bp. The SSC region of *A*. *sieboldii* is reverse-tandemly duplicated and then integrated into two IRs (Supplementary Material 1–3; https://species.nibr.go.kr/gi/search/chloroplastView/WCN0005156.do). The *A*. *sieboldii* genome contains 78 protein-coding genes, 30 tRNA genes, and 4 rRNA genes. The total number of identified genes encoded is 112 genes, with 12 genes duplicated within the SSC integrated into IRs (Supplementary Material 2).

To understand phylogenetic relationship of *A. sieboldii* with other taxa, a neighbor-joining (NJ) tree was constructed using 62 common protein-coding genes of *A. sieboldii* and 25 vascular plant taxa (Figure 1). The NJ tree showed that *A. sieboldii* was grouped with *Piper coenoclatum* belonging to the Piperales order (Figure 1). Chloroplast genome comparison revealed that *A. sieboldii* showed 62.9% similarity with *P. coenoclatum* and had 32,732bp larger genome than *P. coenoclatum* because of extended SC and IRs (Supplementary Materials 1–3).

**Figure 1.**
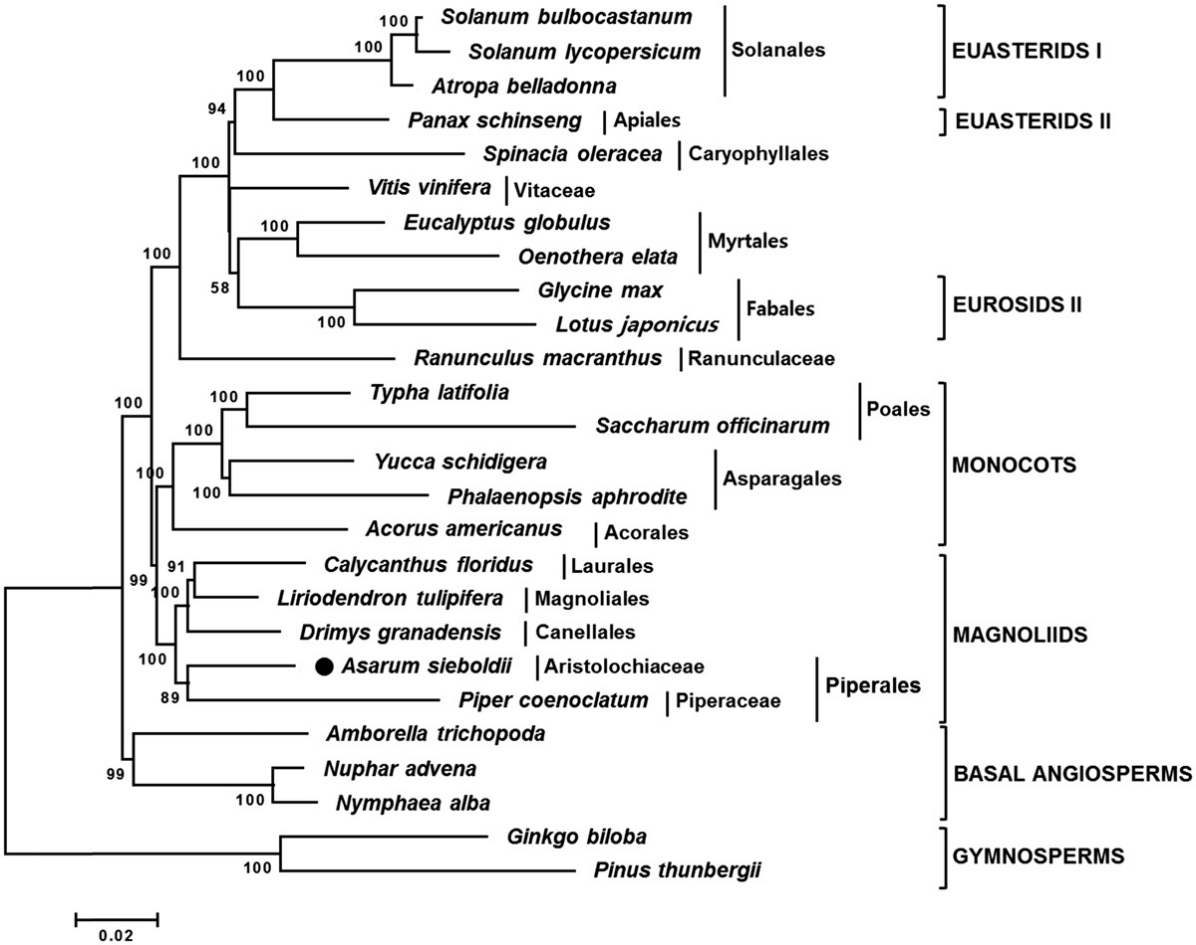
Neighbor-joining (NJ) tree based on the chloroplast protein-coding genes of 26 taxa including *A. sieboldii*. Sequences of 65 chloroplast protein-coding gene from 26 taxa were aligned using MAFFT (http://mafft.cbrc.jp/alignment/server/index.html) and used to generate NJ phylogenetic tree by MEGA 6.0 (Tamura et al. 2013). The numbers in the nodes indicated the bootstrap support values (>50%) from 1000 replicates. Chloroplast genome sequences used for this tree are: *Acorus americanus*, NC_010093; *Amborella trichopoda*, NC_005086; *A. sieboldii*, MG551543; *Atropa belladonna*, NC_004561; *Calycanthus floridus*, NC_004993; *Drimys granatensis*, DQ887676; *Eucalyptus globulus*, NC_008115; *Ginkgo biloba*, NC_016986 (outgroup); *Glycine max*, NC_007942; *Liriodendron tulipifera*, NC_008326; *Lotus japonicus*, NC_002694; *Nuphar advena*, NC_008788; *Nymphaea alba*, NC_006050; *Oenothera elata*, NC_002693; *Panax schinseng*, NC_006290; *Phalaenopsis aphrodite*, NC_007499; *Pinus thunbergii*, NC_001631 (outgroup); *Piper coenoclatum*, DQ887677; *Ranunculus macranthus*, NC_008796; *Saccharum officinarum*, NC_006084; *Solanum bulbocastanum*, NC_007943; *Solanum lycopersicum*, DQ347959; *Spinacia oleracea*, NC_002202; *Typha latifolia*, NC_013823; *Vitis vinifera*, NC_007957; *Yucca schidigera*, NC_032714.

## Supplementary Material

Chae_Eun_Lim_et_al_supplemental_content.zip
