# Supplementary material for: The complete chloroplast genome sequence of Asarum sieboldii Miq. (Aristolochiaceae), a medicinal plant in Korea
Source: Mitochondrial DNA B Resour. 2018 Jan 12;3(1):118–9. doi: 10.1080/23802359.2018.1424577 (PMC7800627; doi:10.1080/23802359.2018.1424577)
Supplement: Chae_Eun_Lim_et_al_supplemental_content.zip [file TMDN_A_1424577_SM2944.zip › Chae Eun Lim et al supplemental content.docx]

**ONLINE SUPPLEMENTARY MATERIAL**

Supplementary Material 1. Structures and sequence comparison of chloroplast genomes of *Asarum sieboldii* (A) and *Piper coenoclatum* (C, GenBank Acc. No. DQ887677). (A, C) Linear maps and structures of chloroplast genomes were drawn using OGDRAW (<http://ogdraw.mpimp-golm.mpg.de/>) and Self-BLASTN searches. (B) Chloroplast genomes of the two species were compared with each other using BLASTN search and a BLAST viewer. Gray bars connecting both genomes indicate homologous sequences.


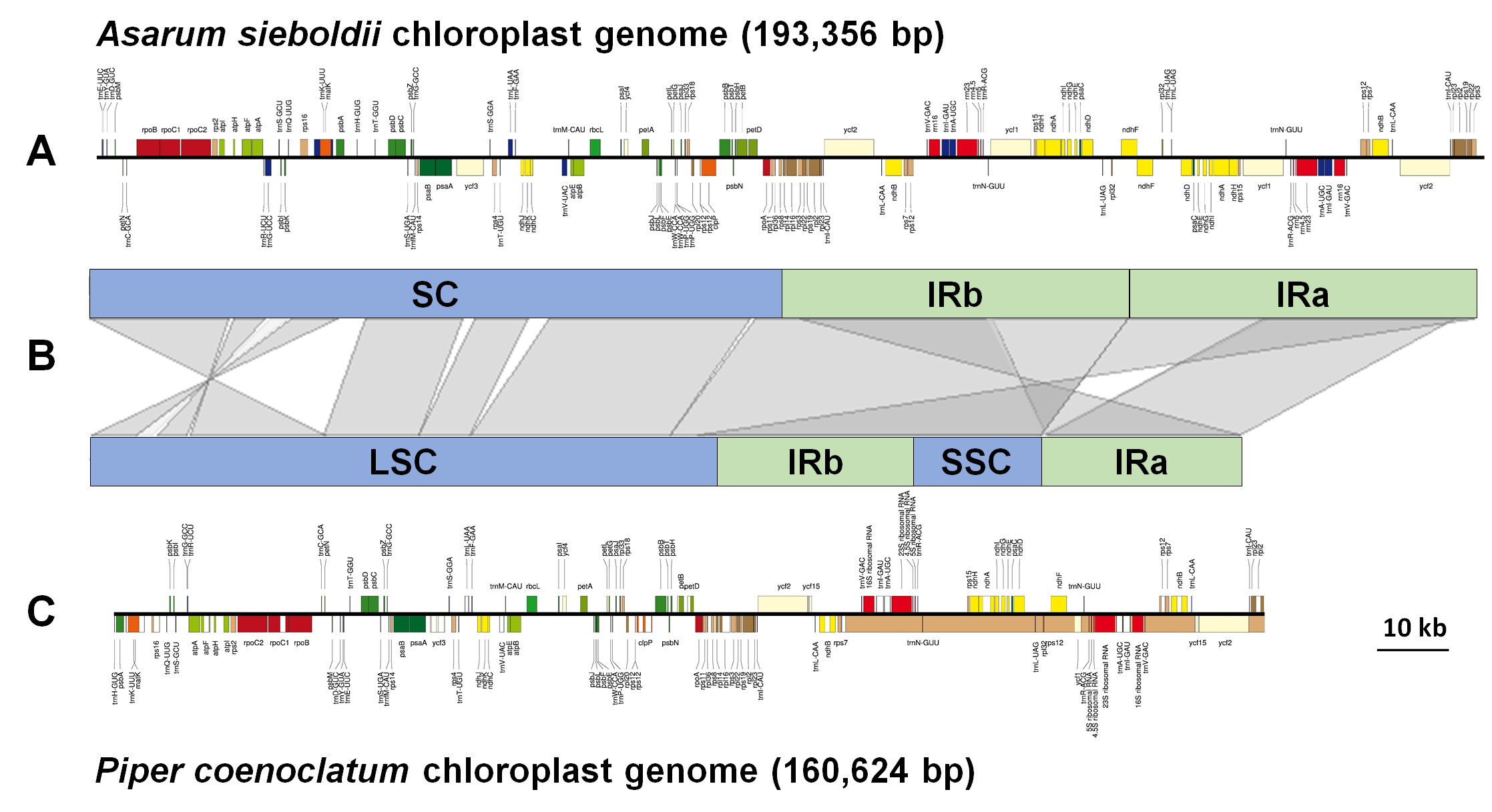


Supplementary Material 2. Gene list of *Asarum sieboldii* chloroplast genome. Genes were annotated using GeSeq (https://chlorobox.mpimp-golm.mpg.de/geseq-app.html) and the Artemis annotation tool (Rutherford et al. 2000).

| **Region** | **Feature** | **Description** | **Start** | **End** | **Strand** | **Exon** |
| --- | --- | --- | --- | --- | --- | --- |
| LSC | trnE-UUC | tRNA-Glu | 724 | 796 | F |  |
| LSC | trnY-GUA | tRNA-Tyr | 861 | 944 | F |  |
| LSC | trnD-GUC | tRNA-Asp | 1402 | 1475 | F |  |
| LSC | psbM | photosystem II protein M | 2523 | 2627 | F |  |
| LSC | petN | cytochrome b6/f complex subunit VIII | 3590 | 3679 | R |  |
| LSC | trnC-GCA | tRNA-Cys | 4130 | 4200 | R |  |
| LSC | rpoB | RNA polymerase beta subunit | 5584 | 8796 | F |  |
| LSC | rpoC1 | RNA polymerase beta' subunit | 8802 | 9254 | F | exon1 |
| LSC | rpoC1 | RNA polymerase beta' subunit | 9995 | 11611 | F | exon2 |
| LSC | rpoC2 | RNA polymerase beta'' subunit | 11773 | 15906 | F |  |
| LSC | rps2 | ribosomal protein S2 | 16114 | 16824 | F |  |
| LSC | atpI | ATP synthase CF0 subunit IV | 17075 | 17818 | F |  |
| LSC | atpH | ATP synthase CF0 subunit III | 19025 | 19270 | F |  |
| LSC | atpF | ATP synthase CF0 subunit I | 20257 | 20401 | F | exon1 |
| LSC | atpF | ATP synthase CF0 subunit I | 21153 | 21562 | F | exon2 |
| LSC | atpA | ATP synthase CF1 alpha subunit | 21631 | 23154 | F |  |
| LSC | trnR-UCU | tRNA-Arg | 23275 | 23346 | R |  |
| LSC | trnG-UCC | tRNA-Gly | 23505 | 23552 | R | exon2 |
| LSC | trnG-UCC | tRNA-Gly | 24287 | 24309 | R | exon1 |
| LSC | trnS-GCU | tRNA-Ser | 25369 | 25456 | F |  |
| LSC | psbI | photosystem II protein I | 25625 | 25759 | R |  |
| LSC | psbK | photosystem II protein K | 26151 | 26336 | R |  |
| LSC | trnQ-UUG | tRNA-Gln | 26666 | 26737 | F |  |
| LSC | rps16 | ribosomal protein S16 | 28352 | 28391 | F | exon1 |
| LSC | rps16 | ribosomal protein S16 | 29233 | 29429 | F | exon2 |
| LSC | trnK-UUU | tRNA-Lys | 30325 | 30361 | F | exon1 |
| LSC | matK | maturase K | 31107 | 32633 | F |  |
| LSC | trnK-UUU | tRNA-Lys | 32904 | 32938 | F | exon2 |
| LSC | psbA | photosystem II protein D1 | 33399 | 34460 | F |  |
| LSC | trnH-GUG | tRNA-His | 36243 | 36317 | F |  |
| LSC | trnT-GGU | tRNA-Thr | 38764 | 38835 | F |  |
| LSC | psbD | photosystem II protein D2 | 40592 | 41653 | F |  |
| LSC | psbC | photosystem II CP43 chlorophyll apoprotein | 41601 | 43022 | F |  |
| LSC | trnS-UGA | tRNA-Ser | 43305 | 43397 | R |  |
| LSC | psbZ | photosystem II protein Z | 43768 | 43956 | F |  |
| LSC | trnG-GCC | tRNA-Gly | 44124 | 44194 | F |  |
| LSC | trnfM-CAU | tRNA-Met | 44317 | 44390 | R |  |
| LSC | rps14 | ribosomal protein S14 | 44552 | 44854 | R |  |
| LSC | psaB | photosystem I P700 apoprotein A2 | 44986 | 47190 | R |  |
| LSC | psaA | photosystem I P700 apoprotein A1 | 47216 | 49468 | R |  |
| LSC | ycf3 | photosystem I assembly protein ycf3 | 50148 | 50300 | R | exon3 |
| LSC | ycf3 | photosystem I assembly protein ycf3 | 51039 | 51268 | R | exon2 |
| LSC | ycf3 | photosystem I assembly protein ycf3 | 53782 | 53905 | R | exon1 |
| LSC | trnS-GGA | tRNA-Ser | 54743 | 54830 | F |  |
| LSC | rps4 | ribosomal protein S4 | 55126 | 55731 | R |  |
| LSC | trnT-UGU | tRNA-Thr | 56174 | 56246 | R |  |
| LSC | trnL-UAA | tRNA-Leu | 57337 | 57371 | F | exon1 |
| LSC | trnL-UAA | tRNA-Leu | 57895 | 57944 | F | exon2 |
| LSC | trnF-GAA | tRNA-Phe | 58303 | 58375 | F |  |
| LSC | ndhJ | NADH-plastoquinone oxidoreductase subunit J | 59091 | 59567 | R |  |
| LSC | ndhK | NADH-plastoquinone oxidoreductase subunit K | 59678 | 60355 | R |  |
| LSC | ndhC | NADH-plastoquinone oxidoreductase subunit 3 | 60415 | 60777 | R |  |
| LSC | trnV-UAC | tRNA-Val | 64824 | 64858 | R | exon2 |
| LSC | trnV-UAC | tRNA-Val | 65502 | 65540 | R | exon1 |
| LSC | trnM-CAU | tRNA-Met | 65723 | 65795 | F |  |
| LSC | atpE | ATP synthase CF1 epsilon subunit | 66029 | 66442 | R |  |
| LSC | atpB | ATP synthase CF1 beta subunit | 66439 | 67935 | R |  |
| LSC | rbcL | ribulose-1,5-bisphosphate carboxylase/oxygenase large subunit | 68746 | 70173 | F |  |
| LSC | accD | acetyl-CoA carboxylase beta subunit | 70904 | 72388 | F |  |
| LSC | psaI | photosystem I subunit VIII | 73012 | 73122 | F |  |
| LSC | ycf4 | photosystem I assembly protein ycf4 | 73498 | 74052 | F |  |
| LSC | petA | cytochrome f | 75964 | 76926 | F |  |
| LSC | psbJ | photosystem II protein J | 77996 | 78118 | R |  |
| LSC | psbL | photosystem II protein L | 78255 | 78371 | R |  |
| LSC | psbF | photosystem II cytochrome b559 beta subunit | 78394 | 78513 | R |  |
| LSC | psbE | photosystem II cytochrome b559 alpha subunit | 78523 | 78774 | R |  |
| LSC | petL | cytochrome b6/f complex subunit VI | 80064 | 80159 | F |  |
| LSC | petG | cytochrome b6/f complex subunit V | 80342 | 80455 | F |  |
| LSC | trnW-CCA | tRNA-Trp | 80588 | 80661 | R |  |
| LSC | trnP-UGG | tRNA-Pro | 80828 | 80901 | R |  |
| LSC | psaJ | photosystem I subunit IX | 81359 | 81493 | F |  |
| LSC | rpl33 | ribosomal protein L33 | 81906 | 82106 | F |  |
| LSC | rps18 | ribosomal protein S18 | 82282 | 82599 | F |  |
| LSC | rpl20 | ribosomal protein L20 | 82872 | 83231 | R |  |
| LSC | rps12 | ribosomal protein S12 | 84033 | 84146 | R | exon1 |
| LSC | clpP | clp protease proteolytic subunit | 84290 | 84535 | R | exon3 |
| LSC | clpP | clp protease proteolytic subunit | 85219 | 85510 | R | exon2 |
| LSC | clpP | clp protease proteolytic subunit | 86240 | 86310 | R | exon1 |
| LSC | psbB | photosystem II CP47 chlorophyll apoprotein | 86769 | 88295 | F |  |
| LSC | psbT | photosystem II protein T | 88486 | 88593 | F |  |
| LSC | psbN | photosystem II protein N | 88664 | 88795 | R |  |
| LSC | psbH | photosystem II phosphoprotein | 88903 | 89124 | F |  |
| LSC | petB | cytochrome b6 | 89244 | 89249 | F | exon1 |
| LSC | petB | cytochrome b6 | 89994 | 90635 | F | exon2 |
| LSC | petD | cytochrome b6/f complex subunit IV | 90826 | 90833 | F | exon1 |
| LSC | petD | cytochrome b6/f complex subunit IV | 91558 | 92047 | F | exon2 |
| LSC | rpoA | RNA polymerase alpha subunit | 92841 | 93848 | R |  |
| LSC | rps11 | ribosomal protein S11 | 93922 | 94338 | R |  |
| LSC | rpl36 | ribosomal protein L36 | 94451 | 94564 | R |  |
| LSC | infA | translation initiation factor 1 | 94680 | 94913 | R |  |
| LSC | rps8 | ribosomal protein S8 | 95035 | 95433 | R |  |
| LSC | rpl14 | ribosomal protein L14 | 95621 | 95989 | R |  |
| LSC | rpl16 | ribosomal protein L16 | 96119 | 96517 | R | exon2 |
| LSC | rpl16 | ribosomal protein L16 | 97528 | 97536 | R | exon1 |
| IRb | rps3 | ribosomal protein S3 | 97692 | 98348 | R |  |
| IRb | rpl22 | ribosomal protein L22 | 98293 | 98835 | R |  |
| IRb | rps19 | ribosomal protein S19 | 98884 | 99162 | R |  |
| IRb | rpl2 | ribosomal protein L2 | 99251 | 99681 | R | exon2 |
| IRb | rpl2 | ribosomal protein L2 | 100347 | 100737 | R | exon1 |
| IRb | rpl23 | ribosomal protein L23 | 100756 | 101058 | R |  |
| IRb | trnI-CAU | tRNA-Ile | 101224 | 101297 | R |  |
| IRb | ycf2 | hypothetical protein RF2 | 101381 | 108301 | F |  |
| IRb | trnL-CAA | tRNA-Leu | 109334 | 109414 | R |  |
| IRb | ndhB | NADH-plastoquinone oxidoreductase subunit 2 | 109928 | 110683 | R | exon2 |
| IRb | ndhB | NADH-plastoquinone oxidoreductase subunit 2 | 111392 | 112168 | R | exon1 |
| IRb | rps7 | ribosomal protein S7 | 112492 | 112959 | R |  |
| IRb | rps12 | ribosomal protein S12 | 113018 | 113043 | R | exon3 |
| IRb | rps12 | ribosomal protein S12 | 113580 | 113811 | R | exon2 |
| IRb | trnV-GAC | tRNA-Val | 115684 | 115755 | F |  |
| IRb | rrn16 | 16S ribosomal RNA | 115983 | 117473 | F |  |
| IRb | trnI-GAU | tRNA-Ile | 117764 | 117800 | F | exon1 |
| IRb | trnI-GAU | tRNA-Ile | 118743 | 118777 | F | exon2 |
| IRb | trnA-UGC | tRNA-Ala | 118842 | 118879 | F | exon1 |
| IRb | trnA-UGC | tRNA-Ala | 119684 | 119718 | F | exon2 |
| IRb | rrn23 | 23S ribosomal RNA | 119871 | 122686 | F |  |
| IRb | rrn4.5 | 4.5S ribosomal RNA | 122785 | 122887 | F |  |
| IRb | rrn5 | 5S ribosomal RNA | 123111 | 123231 | F |  |
| IRb | trnR-ACG | tRNA-Arg | 123484 | 123557 | F |  |
| IRb | trnN-GUU | tRNA-Asn | 124178 | 124249 | R |  |
| IRb | ycf1 | hypothetical protein RF1 | 124567 | 130164 | F |  |
| IRb (SSC tandem-2) | rps15 | ribosomal protein S15 | 130565 | 130837 | F |  |
| IRb (SSC tandem-2) | ndhH | NADH-plastoquinone oxidoreductase subunit 7 | 130936 | 132117 | F |  |
| IRb (SSC tandem-2) | ndhA | NADH-plastoquinone oxidoreductase subunit 1 | 132119 | 132671 | F | exon1 |
| IRb (SSC tandem-2) | ndhA | NADH-plastoquinone oxidoreductase subunit 1 | 133784 | 134322 | F | exon2 |
| IRb (SSC tandem-2) | ndhI | NADH-plastoquinone oxidoreductase subunit I | 134401 | 134916 | F |  |
| IRb (SSC tandem-2) | ndhG | NADH-plastoquinone oxidoreductase subunit 6 | 135249 | 135779 | F |  |
| IRb (SSC tandem-2) | ndhE | NADH-plastoquinone oxidoreductase subunit 4L | 136277 | 136582 | F |  |
| IRb (SSC tandem-2) | psaC | photosystem I subunit VII | 136928 | 137173 | F |  |
| IRb (SSC tandem-2) | ndhD | NADH-plastoquinone oxidoreductase subunit 4 | 137301 | 138812 | F |  |
| IRb (SSC tandem-2) | ccsA | cytochrome c heme attachment protein | 139025 | 139984 | R |  |
| IRb (SSC tandem-2) | trnL-UAG | tRNA-Leu | 140111 | 140190 | R |  |
| IRb (SSC tandem-2) | rpl32 | ribosomal protein L32 | 141349 | 141513 | R |  |
| IRb (SSC tandem-2) | ndhF | NADH-plastoquinone oxidoreductase subunit 5 | 142753 | 144957 | F |  |
| IRa (SSC tandem-1) | ndhF | NADH-plastoquinone oxidoreductase subunit 5 | 144954 | 147158 | R |  |
| IRa (SSC tandem-1) | rpl32 | ribosomal protein L32 | 148398 | 148562 | F |  |
| IRa (SSC tandem-1) | trnL-UAG | tRNA-Leu | 149721 | 149800 | F |  |
| IRa (SSC tandem-1) | ccsA | cytochrome c heme attachment protein | 149927 | 150886 | F |  |
| IRa (SSC tandem-1) | ndhD | NADH-plastoquinone oxidoreductase subunit 4 | 151099 | 152610 | R |  |
| IRa (SSC tandem-1) | psaC | photosystem I subunit VII | 152738 | 152983 | R |  |
| IRa (SSC tandem-1) | ndhE | NADH-plastoquinone oxidoreductase subunit 4L | 153329 | 153634 | R |  |
| IRa (SSC tandem-1) | ndhG | NADH-plastoquinone oxidoreductase subunit 6 | 154132 | 154662 | R |  |
| IRa (SSC tandem-1) | ndhI | NADH-plastoquinone oxidoreductase subunit I | 154995 | 155510 | R |  |
| IRa (SSC tandem-1) | ndhA | NADH-plastoquinone oxidoreductase subunit 1 | 155589 | 156127 | R | exon2 |
| IRa (SSC tandem-1) | ndhA | NADH-plastoquinone oxidoreductase subunit 1 | 157240 | 157792 | R | exon1 |
| IRa (SSC tandem-1) | ndhH | NADH-plastoquinone oxidoreductase subunit 7 | 157794 | 158975 | R |  |
| IRa (SSC tandem-1) | rps15 | ribosomal protein S15 | 159074 | 159346 | R |  |
| IRa | ycf1 | hypothetical protein RF1 | 159747 | 165344 | R |  |
| IRa | trnN-GUU | tRNA-Asn | 165662 | 165733 | F |  |
| IRa | trnR-ACG | tRNA-Arg | 166354 | 166427 | R |  |
| IRa | rrn5 | 5S ribosomal RNA | 166680 | 166800 | R |  |
| IRa | rrn4.5 | 4.5S ribosomal RNA | 167024 | 167126 | R |  |
| IRa | rrn23 | 23S ribosomal RNA | 167225 | 170040 | R |  |
| IRa | trnA-UGC | tRNA-Ala | 170193 | 170227 | R | exon2 |
| IRa | trnA-UGC | tRNA-Ala | 171032 | 171069 | R | exon1 |
| IRa | trnI-GAU | tRNA-Ile | 171134 | 171168 | R | exon2 |
| IRa | trnI-GAU | tRNA-Ile | 172111 | 172147 | R | exon1 |
| IRa | rrn16 | 16S ribosomal RNA | 172438 | 173928 | R |  |
| IRa | trnV-GAC | tRNA-Val | 174156 | 174227 | R |  |
| IRa | rps12 | ribosomal protein S12 | 176100 | 176331 | F | exon2 |
| IRa | rps12 | ribosomal protein S12 | 176868 | 176893 | F | exon3 |
| IRa | rps7 | ribosomal protein S7 | 176952 | 177419 | F |  |
| IRa | ndhB | NADH-plastoquinone oxidoreductase subunit 2 | 177743 | 178519 | F | exon1 |
| IRa | ndhB | NADH-plastoquinone oxidoreductase subunit 2 | 179228 | 179983 | F | exon2 |
| IRa | trnL-CAA | tRNA-Leu | 180497 | 180577 | F |  |
| IRa | ycf2 | hypothetical protein RF2 | 181610 | 188530 | R |  |
| IRa | trnI-CAU | tRNA-Ile | 188614 | 188687 | F |  |
| IRa | rpl23 | ribosomal protein L23 | 188853 | 189155 | F |  |
| IRa | rpl2 | ribosomal protein L2 | 189174 | 189564 | F | exon1 |
| IRa | rpl2 | ribosomal protein L2 | 190230 | 190660 | F | exon2 |
| IRa | rps19 | ribosomal protein S19 | 190749 | 191027 | F |  |
| IRa | rpl22 | ribosomal protein L22 | 191076 | 191618 | F |  |
| IRa | rps3 | ribosomal protein S3 | 191563 | 192219 | F |  |

Supplementary Material 3. Gene list of *Piper coenoclatum* chloroplast genome. Gene information was retrieved from *P. coenoclatum* chloroplast genome (GenBank Acc. No. DQ887677, Cai et al. 2006) deposited in GenBank (<https://www.ncbi.nlm.nih.gov/nuccore/DQ887677>).

| **Region** | **Feature** | **Description** | **Start** | **End** | **Strand** | **Exon** |
| --- | --- | --- | --- | --- | --- | --- |
| LSC | trnH-GUG | tRNA-His | 27 | 101 | R |  |
| LSC | psbA | photosystem II protein D1 | 294 | 1355 | R |  |
| LSC | trnK-UUU | tRNA-Lys | 1652 | 1686 | R | exon2 |
| LSC | matK | maturase K | 1967 | 3505 | R |  |
| LSC | trnK-UUU | tRNA-Lys | 4224 | 4260 | F | exon1 |
| LSC | rps16 | ribosomal protein S16 | 5265 | 5518 | R | exon2 |
| LSC | rps16 | ribosomal protein S16 | 6327 | 6366 | R | exon1 |
| LSC | trnQ-UUG | tRNA-Gln | 7316 | 7388 | R |  |
| LSC | psbK | photosystem II protein K | 7713 | 7892 | F |  |
| LSC | psbI | photosystem II protein I | 8298 | 8408 | F |  |
| LSC | trnS-GCU | tRNA-Ser | 8583 | 8670 | R |  |
| LSC | trnG-GCC | tRNA-Gly | 10160 | 10196 | F |  |
| LSC | trnR-UCU | tRNA-Arg | 10255 | 10326 | F |  |
| LSC | atpA | ATP synthase CF1 alpha subunit | 10436 | 11959 | R |  |
| LSC | atpF | ATP synthase CF0 subunit I | 12111 | 12520 | R | exon2 |
| LSC | atpF | ATP synthase CF0 subunit I | 13261 | 13405 | R | exon1 |
| LSC | atpH | ATP synthase CF0 subunit III | 13958 | 14203 | R |  |
| LSC | atpI | ATP synthase CF0 subunit IV | 15303 | 16046 | R |  |
| LSC | rps2 | ribosomal protein S2 | 16294 | 17004 | R |  |
| LSC | rpoC2 | RNA polymerase beta'' subunit | 17256 | 21398 | R |  |
| LSC | rpoC1 | RNA polymerase beta' subunit | 21550 | 23184 | R | exon2 |
| LSC | rpoC1 | RNA polymerase beta' subunit | 23956 | 24387 | R | exon1 |
| LSC | rpoB | RNA polymerase beta subunit | 24414 | 27626 | R |  |
| LSC | trnC-GCA | tRNA-Cys | 28924 | 29004 | F |  |
| LSC | petN | cytochrome b6/f complex subunit VIII | 29393 | 29482 | F |  |
| LSC | psbM | photosystem II protein M | 30419 | 30523 | R |  |
| LSC | trnD-GUC | tRNA-Asp | 31545 | 31618 | R |  |
| LSC | trnY-GUA | tRNA-Tyr | 31943 | 32026 | R |  |
| LSC | trnE-UUC | tRNA-Glu | 32086 | 32157 | R |  |
| LSC | trnT-GGU | tRNA-Thr | 32885 | 32956 | F |  |
| LSC | psbD | photosystem II protein D2 | 34486 | 35550 | F |  |
| LSC | psbC | photosystem II CP43 chlorophyll apoprotein | 35498 | 36919 | F |  |
| LSC | trnS-UGA | tRNA-Ser | 37171 | 37263 | R |  |
| LSC | psbZ | photosystem II protein Z | 37625 | 37813 | F |  |
| LSC | trnG-GCC | tRNA-Gly | 38104 | 38174 | F |  |
| LSC | trnfM-CAU | tRNA-Met | 38343 | 38416 | R |  |
| LSC | rps14 | ribosomal protein S14 | 38574 | 38876 | R |  |
| LSC | psaB | photosystem I P700 apoprotein A2 | 39015 | 41219 | R |  |
| LSC | psaA | photosystem I P700 apoprotein A1 | 41245 | 43497 | R |  |
| LSC | ycf3 | photosystem I assembly protein ycf3 | 44150 | 44302 | R | exon3 |
| LSC | ycf3 | photosystem I assembly protein ycf3 | 45033 | 45262 | R | exon2 |
| LSC | ycf3 | photosystem I assembly protein ycf3 | 46086 | 46209 | R | exon1 |
| LSC | trnS-GGA | tRNA-Ser | 46741 | 46827 | F |  |
| LSC | rps4 | ribosomal protein S4 | 47115 | 47720 | R |  |
| LSC | trnT-UGU | tRNA-Thr | 48090 | 48162 | R |  |
| LSC | trnL-UAA | tRNA-Leu | 48970 | 49004 | F | exon1 |
| LSC | trnL-UAA | tRNA-Leu | 49495 | 49544 | F | exon2 |
| LSC | trnF-GAA | tRNA-Phe | 49928 | 50000 | F |  |
| LSC | ndhJ | NADH-plastoquinone oxidoreductase subunit J | 50750 | 51226 | R |  |
| LSC | ndhK | NADH-plastoquinone oxidoreductase subunit K | 51339 | 52208 | R |  |
| LSC | ndhC | NADH-plastoquinone oxidoreductase subunit 3 | 52088 | 52450 | R |  |
| LSC | trnV-UAC | tRNA-Val | 53796 | 53829 | R | exon2 |
| LSC | trnV-UAC | tRNA-Val | 54431 | 54469 | R | exon1 |
| LSC | trnM-CAU | tRNA-Met | 54627 | 54699 | F |  |
| LSC | atpE | ATP synthase CF1 epsilon subunit | 54921 | 55322 | R |  |
| LSC | atpB | ATP synthase CF1 beta subunit | 55319 | 56815 | R |  |
| LSC | rbcL | ribulose-1,5-bisphosphate carboxylase/oxygenase large subunit | 57610 | 59037 | F |  |
| LSC | accD | acetyl-CoA carboxylase beta subunit | 59799 | 61325 | F |  |
| LSC | psaI | photosystem I subunit VIII | 62028 | 62138 | F |  |
| LSC | ycf4 | photosystem I assembly protein ycf4 | 62606 | 63160 | F |  |
| LSC | cemA | chloroplast envelope membrane protein | 64190 | 64879 | F |  |
| LSC | petA | cytochrome f | 65109 | 66071 | F |  |
| LSC | psbJ | photosystem II protein J | 66931 | 67053 | R |  |
| LSC | psbL | photosystem II protein L | 67177 | 67293 | R |  |
| LSC | psbF | photosystem II cytochrome b559 beta subunit | 67316 | 67435 | R |  |
| LSC | psbE | photosystem II cytochrome b559 alpha subunit | 67445 | 67696 | R |  |
| LSC | petL | cytochrome b6/f complex subunit VI | 68773 | 68868 | F |  |
| LSC | petG | cytochrome b6/f complex subunit V | 69067 | 69180 | F |  |
| LSC | trnW-CCA | tRNA-Trp | 69303 | 69376 | R |  |
| LSC | trnP-UGG | tRNA-Pro | 69556 | 69630 | R |  |
| LSC | psaJ | photosystem I subunit IX | 70031 | 70165 | F |  |
| LSC | rpl33 | ribosomal protein L33 | 70570 | 70776 | F |  |
| LSC | rps18 | ribosomal protein S18 | 70955 | 71263 | F |  |
| LSC | rpl20 | ribosomal protein L20 | 71484 | 71870 | R |  |
| LSC | rps12 | ribosomal protein S12 | 72699 | 72812 | R | exon1 |
| LSC | clpP | clp protease proteolytic subunit | 72974 | 73218 | R | exon3 |
| LSC | clpP | clp protease proteolytic subunit | 73857 | 74149 | R | exon2 |
| LSC | clpP | clp protease proteolytic subunit | 75023 | 75093 | R | exon1 |
| LSC | psbB | photosystem II CP47 chlorophyll apoprotein | 75567 | 77093 | F |  |
| LSC | psbT | photosystem II protein T | 77272 | 77373 | F |  |
| LSC | psbN | photosystem II protein N | 77449 | 77580 | R |  |
| LSC | psbH | photosystem II phosphoprotein | 77693 | 77914 | F |  |
| LSC | petB | cytochrome b6 | 78640 | 78645 | F | exon1 |
| LSC | petB | cytochrome b6 | 78867 | 79508 | F | exon2 |
| LSC | petD | cytochrome b6/f complex subunit IV | 80053 | 80058 | F | exon1 |
| LSC | petD | cytochrome b6/f complex subunit IV | 80462 | 80935 | F | exon2 |
| LSC | rpoA | RNA polymerase alpha subunit | 81151 | 82170 | R |  |
| LSC | rps11 | ribosomal protein S11 | 82238 | 82654 | R |  |
| LSC | rpl36 | ribosomal protein L36 | 82781 | 82894 | R |  |
| LSC | infA | translation initiation factor 1 | 83010 | 83243 | R |  |
| LSC | rps8 | ribosomal protein S8 | 83365 | 83769 | R |  |
| LSC | rpl14 | ribosomal protein L14 | 83996 | 84364 | R |  |
| LSC | rpl16 | ribosomal protein L16 | 84511 | 84909 | R | exon2 |
| LSC | rpl16 | ribosomal protein L16 | 86008 | 86016 | R | exon1 |
| LSC | rps3 | ribosomal protein S3 | 86149 | 86811 | R |  |
| LSC | rpl22 | ribosomal protein L22 | 86899 | 87330 | R |  |
| LSC | rps19 | ribosomal protein S19 | 87379 | 87657 | R |  |
| IRb | rpl2 | ribosomal protein L2 | 87796 | 88226 | R | exon2 |
| IRb | rpl2 | ribosomal protein L2 | 88892 | 89282 | R | exon1 |
| IRb | rpl23 | ribosomal protein L23 | 89301 | 89582 | R |  |
| IRb | trnI-CAU | tRNA-Ile | 89748 | 89821 | R |  |
| IRb | ycf2 | hypothetical protein RF2 | 89883 | 96827 | F |  |
| IRb | ycf15 | hypothetical chloroplast RF15, Ycf15 protein | 96883 | 97035 | F | exon1 |
| IRb | ycf15 | hypothetical chloroplast RF15, Ycf15 protein | 97335 | 97448 | F | exon2 |
| IRb | trnL-CAA | tRNA-Leu | 97828 | 97908 | R |  |
| IRb | ndhB | NADH-plastoquinone oxidoreductase subunit 2 | 98478 | 99233 | R | exon2 |
| IRb | ndhB | NADH-plastoquinone oxidoreductase subunit 2 | 99934 | 100710 | R | exon1 |
| IRb | rps7 | ribosomal protein S7 | 101032 | 101499 | R |  |
| IRb | rps12 | ribosomal protein S12 | 101558 | 101583 | R | exon3 |
| IRb | rps12 | ribosomal protein S12 | 102120 | 102351 | R | exon2 |
| IRb | trnV-GAC | tRNA-Val | 104330 | 104401 | F |  |
| IRb | trnI-GAU | tRNA-Ile | 106415 | 106451 | F | exon1 |
| IRb | trnI-GAU | tRNA-Ile | 107397 | 107431 | F | exon2 |
| IRb | trnA-UGC | tRNA-Ala | 107496 | 107533 | F | exon1 |
| IRb | trnA-UGC | tRNA-Ala | 108341 | 108375 | F | exon2 |
| IRb | trnR-ACG | tRNA-Arg | 112154 | 112227 | F |  |
| IRb | trnN-GUU | tRNA-Asn | 112838 | 112909 | R |  |
| SSC | rps15 | ribosomal protein S15 | 119136 | 119408 | F |  |
| SSC | ndhH | NADH-plastoquinone oxidoreductase subunit 7 | 119522 | 120703 | F |  |
| SSC | ndhA | NADH-plastoquinone oxidoreductase subunit 1 | 120705 | 121256 | F | exon1 |
| SSC | ndhA | NADH-plastoquinone oxidoreductase subunit 1 | 122356 | 122895 | F | exon2 |
| SSC | ndhI | NADH-plastoquinone oxidoreductase subunit I | 122974 | 123516 | F |  |
| SSC | ndhG | NADH-plastoquinone oxidoreductase subunit 6 | 123886 | 124416 | F |  |
| SSC | ndhE | NADH-plastoquinone oxidoreductase subunit 4L | 124649 | 124954 | F |  |
| SSC | psaC | photosystem I subunit VII | 125259 | 125504 | F |  |
| SSC | ndhD | NADH-plastoquinone oxidoreductase subunit 4 | 125622 | 127118 | F |  |
| SSC | ccsA | cytochrome c heme attachment protein | 127456 | 128421 | R |  |
| SSC | trnL-UAG | tRNA-Leu | 128566 | 128645 | R |  |
| SSC | rpl32 | ribosomal protein L32 | 129680 | 129844 | R |  |
| SSC | ndhF | NADH-plastoquinone oxidoreductase subunit 5 | 130771 | 132993 | F |  |
| SSC | ycf1 | hypothetical protein RF1 | 134099 | 135025 | R |  |
| IRa | trnN-GUU | tRNA-Asn | 135384 | 135455 | F |  |
| IRa | trnR-ACG | tRNA-Arg | 136066 | 136139 | R |  |
| IRa | trnA-UGC | tRNA-Ala | 139917 | 139951 | R | exon2 |
| IRa | trnA-UGC | tRNA-Ala | 140760 | 140797 | R | exon1 |
| IRa | trnI-GAU | tRNA-Ile | 140862 | 140896 | R | exon2 |
| IRa | trnI-GAU | tRNA-Ile | 141842 | 141878 | R | exon1 |
| IRa | trnV-GAC | tRNA-Val | 143892 | 143963 | R |  |
| IRa | rps12 | ribosomal protein S12 | 145942 | 146173 | F | exon2 |
| IRa | rps12 | ribosomal protein S12 | 146710 | 146735 | F | exon3 |
| IRa | rps7 | ribosomal protein S7 | 146794 | 147261 | F |  |
| IRa | ndhB | NADH-plastoquinone oxidoreductase subunit 2 | 147583 | 148359 | F | exon1 |
| IRa | ndhB | NADH-plastoquinone oxidoreductase subunit 2 | 149060 | 149815 | F | exon2 |
| IRa | trnL-CAA | tRNA-Leu | 150385 | 150465 | F |  |
| IRa | ycf15 | hypothetical chloroplast RF15, Ycf15 protein | 150845 | 150958 | R | exon2 |
| IRa | ycf15 | hypothetical chloroplast RF15, Ycf15 protein | 151258 | 151410 | R | exon1 |
| IRa | ycf2 | hypothetical protein RF2 | 151466 | 158410 | R |  |
| IRa | trnI-CAU | tRNA-Ile | 158472 | 158545 | F |  |
| IRa | rpl23 | ribosomal protein L23 | 158711 | 158992 | F |  |
| IRa | rpl2 | ribosomal protein L2 | 159011 | 159401 | F | exon1 |
| IRa | rpl2 | ribosomal protein L2 | 160067 | 160497 | F | exon2 |

**References**

Rutherford K, Parkhill J, Crook J, Horsnell T, Rice P, Rajandream MA, Barrell B. 2000. Artemis: sequence visualization and annotation. Bioinformatics. 16:944-945

Cai Z, Penaflor C, Kuehl JV, Leebens-Mack J, Carlson JE, dePamphilis CW, Boore JL, Jansen RK. 2006. Complete plastid genome sequences of Drimys, Liriodendron, and Piper: implications for the phylogenetic relationships of magnoliids. BMC Evol Biol. 6:77.
